# Proton pump inhibitors and hypomagnesemia: A meta-analysis of observational studies

**DOI:** 10.1097/MD.0000000000017788

**Published:** 2019-11-01

**Authors:** Thawin Srinutta, Api Chewcharat, Kullaya Takkavatakarn, Kearkiat Praditpornsilpa, Somchai Eiam-Ong, Bertrand L. Jaber, Paweena Susantitaphong

**Affiliations:** a Division of Nephrology, Department of Medicine, King Chulalongkorn Memorial Hospital, Faculty of Medicine, Chulalongkorn University, Bangkok, Thailand; b Department of Medicine, St. Elizabeth's Medical Center; c Department of Medicine, Tufts University School of Medicine, Boston, MA; d Research Unit for Metabolic Bone Disease in CKD patients, Chulalongkorn University, Bangkok, Thailand.

**Keywords:** hypomagnesemia, meta-analysis, PPI, proton pump inhibitor, systematic review

## Abstract

**Background:**

Previous meta-analyses have suggested that there might be an association between the use of proton pump inhibitors (PPIs) and the development of hypomagnesemia, although the conclusions were no definitive.

**Methods:**

To provide an update on this topic, we performed a meta-analysis of all observational studies that examined the association between the use of PPIs and the development of hypomagnesemia. A literature search was conducted in MEDLINE, Scopus and Cochrane Central Register of Controlled Trials (January 1970 to June 2018) to identify observational studies that examined the association between the use of PPIs and the incidence and prevalence of hypomagnesemia.

**Study eligibility criteria:**

In the absence of randomized controlled trials, we focused primarily on observational studies, including cross-sectional, case-control, retrospective, and prospective cohort studies. There was no limitation on sample size or study duration. Random-effect models meta-analyses were used to compute pooled unadjusted and adjusted odds ratios (ORs) for binary variables.

**Results:**

Sixteen observational studies were identified, including 13 cross-sectional studies, 2 case-control studies, and 1 cohort study, with a total of 131,507 patients. The pooled percentage of PPI users was 43.6% (95% confidence interval [CI] 25.0%, 64.0%). Among PPI users, 19.4% (95% CI 13.8%, 26.5%) had hypomagnesemia compared to 13.5% (95% CI 7.9%, 22.2%) among nonusers. By meta-analysis, PPI use was significantly associated with hypomagnesemia, with a pooled unadjusted OR of 1.83 (95% CI 1.26, 2.67; *P* = .002) and a pooled adjusted OR of 1.71 (95% CI 1.33, 2.19; *P* < .001). In subgroup analyses, high-dose PPI use was associated with higher odds for hypomagnesemia relative to low-dose PPI use (pooled adjusted OR 2.13; 95% CI 1.26, 3.59; *P* = .005).

**Conclusion:**

Our findings are in support of the results of the previous meta-analyses. Furthermore, we found a dose-response between the PPI use and development of hypomagnesemia.

## Abstract 2

Supplemental Digital Content is available in the text

## 1 Introduction

Proton pump inhibitors (PPIs) are widely used for the treatment of gastroesophageal reflux disease, peptic ulcer disease, and conditions associated with increased gastric acid secretion, and for the prevention of gastric ulcers in patients requiring prolonged use of nonsteroidal anti-inflammatory drugs or corticosteroids.^[1]^ Although the recommended treatment duration is 4 to 8 weeks for acute gastric and duodenal ulcers,^[2]^ the US Food and Drug Administration (FDA) advises that not greater than three 2-week treatment courses per year should be prescribed.^[3]^ High dose and prolonged use (>8 weeks) of PPIs has been linked to an increased risk of *Clostridium difficile* infection,^[4]^ hospital-acquired pneumonia,^[5]^ bone loss, fractures,^[6]^ and mortality.^[7]^

In 2006, an association between the use of PPIs and hypomagnesemia was first described,^[8]^ which was followed by several additional reports.^[9]^ In 2011, the FDA issued a drug safety communication stating that low magnesium levels could be associated with long-term use of PPIs (FDA website. http://www.fda.gov/Drugs/DrugSafety/ucm245011.htm. Accessed October 07, 2018). This safety communication was based on the review of 38 cases from the Adverse Event Reporting System and 23 published case reports. While this information was added to the warnings and precautions sections of the labels for all PPIs, this decision by the FDA was not based on large observational or confirmatory studies. PPIs may cause hypomagnesemia by decreasing intestinal magnesium absorption resulting in decreased urinary magnesium excretion.^[10,11]^ Intestinal absorption of magnesium occurs through a passive and active transport mechanism involving 2 proteins located on the apical membrane of enterocytes, the transient receptor potential melastatin (TRPM) 6 and TRMP7.^[12]^ These proteins have a high affinity for magnesium absorption and play role in maintenance of magnesium balance during periods of sparse dietary magnesium intake.^[12]^ TRPM activity is regulated by the intra-luminal acid-base status whereby an acidic milieu increases its activity.^[13]^ PPIs decrease the activity of TRPM6, resulting in a decrease in intestinal absorption of magnesium and hypomagnesemia.^[13,14]^

Previous observational studies^[15,16]^ have demonstrated variable associations between PPI use and hypomagnesemia. Three previously published meta-analyses^[17–19]^ of observational studies have concluded that there might be an association between PPI use and hypomagnesemia. However, some of these reports did not conduct adequate adjustment for confounding factors. To provide an update on this topic, we performed a meta-analysis of all observational studies that examined this question, and explored whether there was an association between PPI dose or treatment duration and the development of hypomagnesemia.

## 2 Methods

### 2.1 Data sources and searches

The review was conducted according to the preferred reporting items for systematic reviews and meta-analyses statement. In brief, we conducted electronic searches in MEDLINE, Scopus, and Cochrane Central Register of Controlled Trials (1970 through June 2018) to identify eligible studies using the medical subject headings database search terms “proton pump inhibitor,” or “omeprazole,” or “esomeprazole,” or “lansoprazole,” or “dexlansoprazole,” or “pantoprazole,” or “rabeprazole,” and “magnesium.” We also searched ClinicalTrials.gov. The search was limited to the English language and focused on human studies.

### 2.2 Study selection

In the absence of randomized controlled trials, we focused primarily on observational studies, including cross-sectional, case-control, retrospective, and prospective cohort studies, which examined the association between PPI use and presence (prevalence) or development (incidence) of hypomagnesemia. There was no limitation on sample size or study duration.

### 2.3 Data extraction and quality assessment

Data were extracted in duplicate by 2 authors (TS and AC), and disagreements were resolved through consensus and arbitration by a third author (PS). The following study-level characteristics were extracted: author's last name, country of origin, year of publication, study design, sample size, population setting, definition of hypomagnesemia, and exclusion criteria. The following patient-level summary characteristics were extracted: mean age, percentage of women, percentage with diabetes mellitus, percentage using diuretics, percentage using PPIs, type, dose and treatment duration of PPIs, and mean baseline serum creatinine and serum magnesium level.

For the 2 outcomes of interest, presence of hypomagnesemia (binary outcome variable) and serum magnesium level (continuous outcome variable), we extracted data on the number and percentage of patients who had hypomagnesemia. If available, we also extracted data on hypomagnesemia-associated adverse events (eg, cardiac arrhythmias). For the studies that performed multivariable logistic regression analyses, we extracted the unadjusted and adjusted odds ratio (OR) with the corresponding 95% confidence interval (CI) for development of hypomagnesemia among patients taking PPIs relative to those not taking the drug. Covariates used in the multivariable regression analyses were also extracted to improve the interpretation of the strength of these associations and to assess for residual confounding.

The quality of the observational studies was assessed using an adaptation of the National Heart, Lung, and Blood Institute (NHLBI) Study Quality Assessment Tool,^[20]^ with a maximum score of 14 for cross-sectional and cohort studies, and a maximum score of 12 for case-control studies. Studies with a score of 0 to 4, 5 to 9, and >9 were considered of low, fair, and good quality, respectively. Since this was a systematic review of the literature, no institutional review board approval was required.

### 2.4 Data synthesis and analysis

The results of the systematic review were tabulated and synthesized qualitatively. For a subset of studies with analyzable and comparable data, the results were synthesized quantitatively by performing random-effects model meta-analyses to compute absolute net changes in continuous variables (ie, serum magnesium level) and pooled OR for binary variables (ie, presence versus absence of hypomagnesemia). All pooled estimates were displayed with a 95% CI. Existence of heterogeneity among effect sizes of individual studies was assessed using the *Q* test and the *I*^2^ index, with a value of 75% or greater indicating medium-to-high heterogeneity. To explore sources of heterogeneity, we performed subgroup meta-analyses according to PPI dose (high-dose vs low-dose) and population setting (ambulatory, hospital, vs dialysis unit setting).

Publication bias was formally assessed using funnel plots and the Egger test. The analyses were performed using Comprehensive Meta-Analysis version 2.0 (www.meta-analysis.com; Biostat, Englewood, NJ).

## 3 Results

### 3.1 Characteristics of the studies

**Figure 1:**
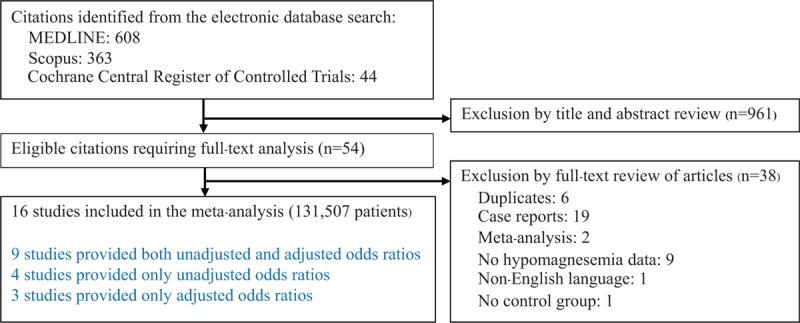
Flow diagram of study selection.

Figure 1 displays the study selection flow diagram. In brief, a total of 1015 potentially relevant citations were identified and screened. Fifty-four citations were evaluated in detail and 38 studies were excluded as they did not meet the inclusion criteria. Sixteen studies fulfilled the inclusion criteria and were included in the systematic review and meta-analysis.

The characteristics of the individual studies are shown in Tables 1 and 2. There were 13 cross-sectional studies,^[21–30,32–34]^ 2 case-control studies,^[15,31]^ and 1 cohort study^[16]^ with a total of 131,507 patients. Seven studies originated from North America,^[15,21–23,31,32,34]^ 6 studies from Europe,^[16,25,26,29,30,33]^ and 3 studies from Asia.^[24,27,28]^ The studies were published between 2012 and 2018 and varied in sample size (62–95,205 patients). The duration of follow-up in the 1 cohort study was 12 months. Four studies involved patients in ambulatory settings,^[16,24,25,30]^ 3 studies in dialysis facilities,^[27,32,33]^ and 9 studies in hospital settings.^[15,21–23,26,28,29,31,34]^ Hypomagnesemia was defined based on a serum magnesium of less than 1.7 mg/dL in 6 studies,^[15,23–25,29,30]^ a serum magnesium of less than 1.6 mg/dL in 4 studies,^[16,21,28,34]^ a serum magnesium of less than 1.8 mg/dL in 3 studies,^[22,26,32]^ a serum magnesium of less than 2.0 mg/dL in 1 study,^[27]^ and a serum magnesium of less than 2.18 mg/dL in 1 study.^[33]^ One study defined hypomagnesemia based on the presence of a diagnosis code of hypomagnesemia, using the 10th Edition, International Classification of Disease, Clinical Modification.^[31]^ The pooled percentage of PPI users was 43.6% (95% CI 25.0%, 64.0%). Different PPIs were used, and doses were variably reported in very few studies, including a defined daily dose (which is the assumed average maintenance dose per day for a PPI used for its main indication), an omeprazole equivalent dose, and a high- versus low-dose.

**Table 1:**
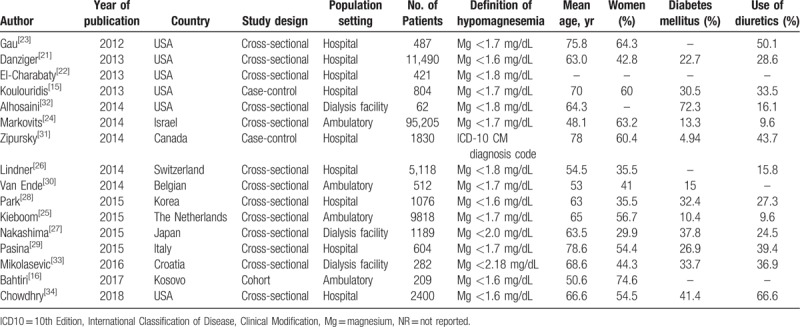
Characteristics of the studies included in the systematic review.

**Table 2:**
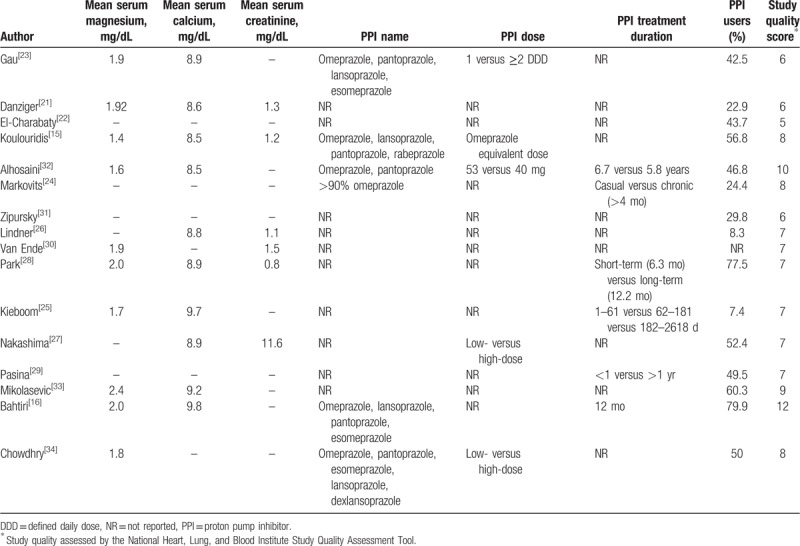
Characteristics of the studies included in the systematic review.

Table 3 displays patient characteristics according to PPI use. The pooled mean age (in years) was 63.8 among PPI users and 62.8 among nonusers, and the pooled percentage of women was 50.4% (95% CI 41.8%, 59.0%) and 44.9% (95% CI 36.9%, 53.1%), respectively. Among PPI users, the pooled estimate percentage of patients taking diuretics was 33.7% (95% CI 21.0%, 49.1%) compared to 30.0% (95% CI 15.3%, 50.6%) among nonusers, and the pooled percentage of patients with diabetes mellitus was 30.6% (95% CI 23.2%, 39.3%) and 27.8% (95% CI 17.3%, 41.4%), respectively. Among PPI users, 19.4% (95% CI 13.8%, 26.5%) had hypomagnesemia compared to 13.5% (95% CI 7.9%, 22.2%) among nonusers.

**Table 3:**
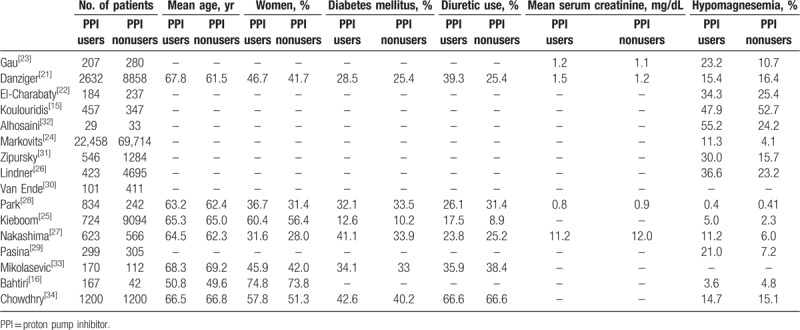
Characteristics of proton pump inhibitor (PPI) users and nonusers in the studies included in the systematic review.

### 3.2 Quality assessment

Using the NHLBI Study Quality Assessment Tool, the quality of the studies was considered as fair to good, with none rated as poor (see Table, Supplemental Digital Content 1, which illustrates the quality scoring for Observational Cohort and Cross-Sectional Studies, Supplemental Digital Content 2, which illustrates the quality scoring of Case-Control Studies).

### 3.3 Association between the use of PPIs and hypomagnesemia

Table 4 summarizes the adjusted OR for hypomagnesemia among PPI users relative to nonusers in the 12 studies that performed multivariable logistic regression analyses. Eight of the 12 studies observed an association between PPI use and hypomagnesemia, and these analyses used a number of covariates in the regression models, including age, sex, comorbidity, concurrent use of drugs potentially affecting serum magnesium levels, and dialysis-related factors (among patients with end-stage renal disease).

**Table 4:**
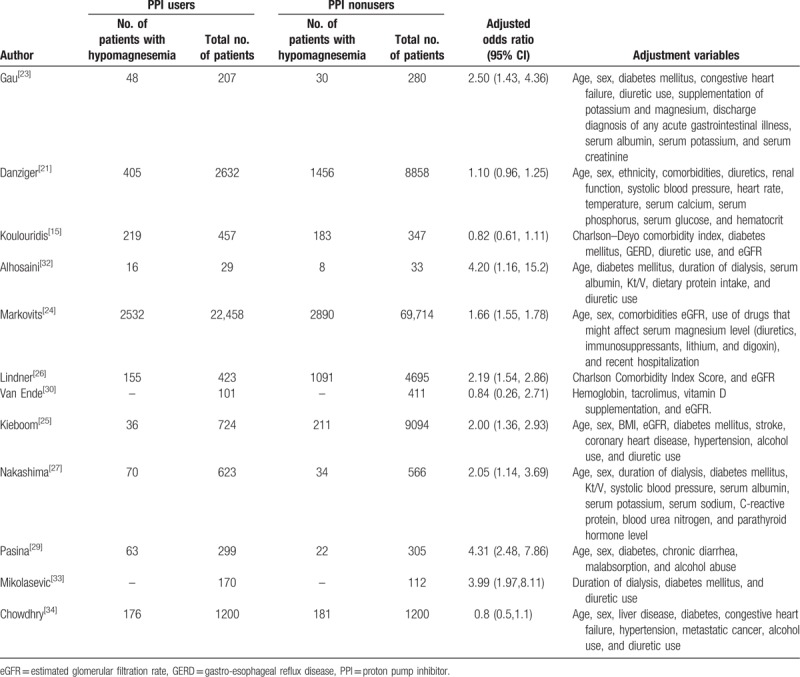
Adjusted odds ratio for hypomagnesemia among proton pump inhibitor users relative to nonusers in the studies included in the meta-analysis.

**Figure 2:**
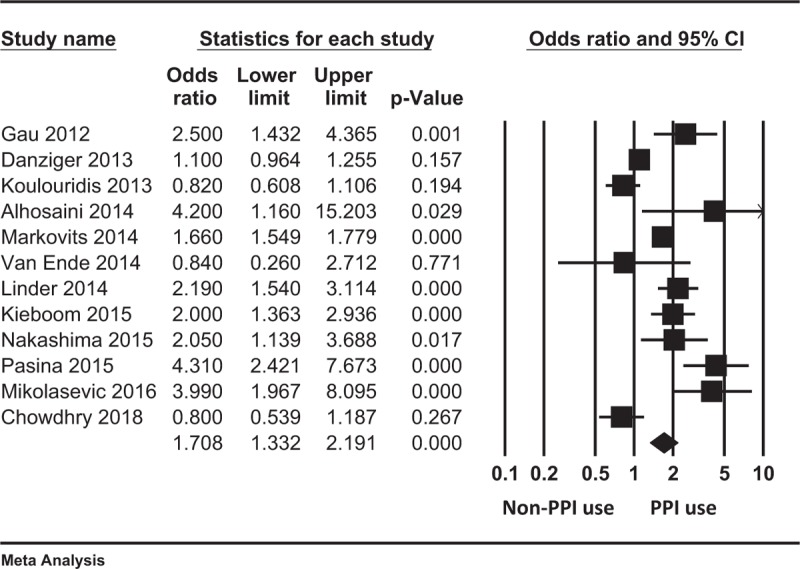
Forest plot displaying the pooled adjusted odds ratio for hypomagnesemia among proton pump inhibitor users relative to nonusers.

There was a significant association between PPI use and hypomagnesemia in both the unadjusted and adjusted analyses (Table 5). Indeed, pooled unadjusted OR for hypomagnesemia was 1.83 (95% CI 1.26, 2.67; *P* = .002) among PPI users (relative to nonusers), and the pooled adjusted OR was 1.71 (95% CI 1.33, 2.19; *P* < .001; Fig. 2). However, there was significant heterogeneity based on the *Q*-test *P*-value and *I*^2^ index (Table 5).

**Table 5:**
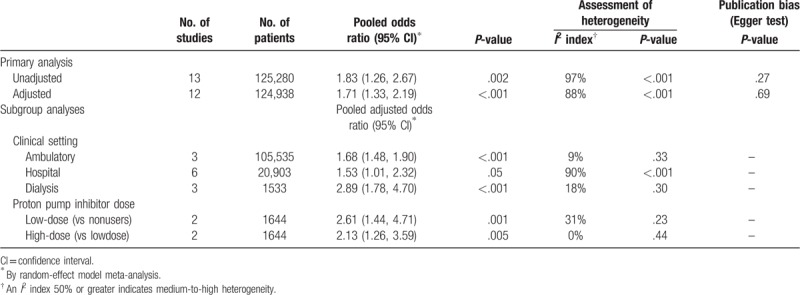
Primary analysis and subgroup analyses examining the association between use of proton pump inhibitors and hypomagnesemia.

Results of the subgroup analyses aimed at exploring sources of heterogeneity are shown in Table 5. In brief, there was a significant association between PPI use and development of hypomagnesemia in ambulatory care settings (pooled adjusted OR 1.68; 95% CI 1.48, 1.90; *P* < .001; 3 studies, 105,535 analyzable patients), in dialysis facilities (pooled adjusted OR 2.89; 95% CI 1.78, 4.70; *P* < .001; 3 studies, 1533 analyzable patients), and in hospital settings (pooled adjusted OR 1.53; 95% CI 1.01, 2.32; *P* = .046; 6 studies, 20,903 analyzable patients).

Patients taking high-dose PPIs had a higher odds of hypomagnesemia relative to those taking low-dose PPIs (pooled adjusted OR 2.13; 95% CI 1.26, 3.59; *P* = .005; 2 studies, 1644 analyzable patients). Furthermore, patients taking low-dose PPIs also had higher odds of hypomagnesemia relative to non-users (pooled adjusted OR 2.61; 95% CI 1.44, 4.71; *P* = .001; 2 studies, 1644 analyzable patients).

### 3.4 Assessment of publication bias

**Figure 3:**
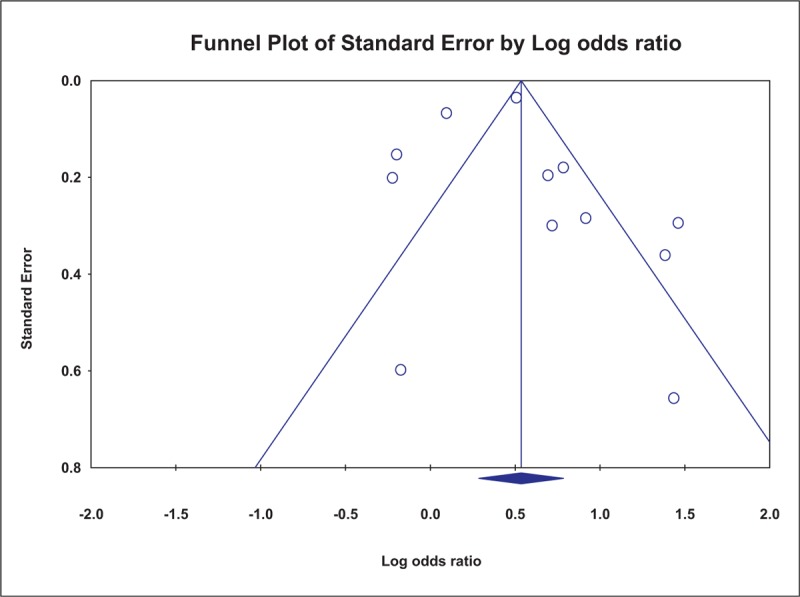
Funnel plot of individual studies displaying the standard error by the log odds ratio for hypomagnesemia among proton pump inhibitor users (relative to nonusers). *P* = .66 by the Egger test.

The funnel plot for the outcome of hypomagnesemia in the studies included in the meta-analysis was symmetric (Fig. 3) and the Egger test was not significant (*P* = .66), suggesting less susceptibility to publication bias.

## 4 Discussion

In the present systematic review and meta-analysis of observational studies, we summarize the existing literature on the association between PPI use and development of hypomagnesemia. Table 6 illustrates the summary of findings from 4 meta-analyses on the association between the use of PPIs and hypomagnesemia. There are 3 previous meta-analyses on this topic (2 that included 9 studies^[17,18]^ and 1 that included 14 studies^[19]^). Some of these reports did not properly account for factors that might confound this association. In addition, in the previously published meta-analyses, while subgroup analyses were conducted according to clinical settings (ambulatory- versus hospital-setting),^[17,19]^ serum magnesium cut-off values^[17,19]^ and study design,^[19]^ none explored the potential association between dose of PPIs and duration of use, and development of hypomagnesemia. We found that low-dose PPI use was associated with increased odds for hypomagnesemia relative to non-PPI use, and that high-dose PPI use was also associated with increased odds for hypomagnesemia relative to low-dose PPI use. Of note, in a recently published prospective open-label comparative study, long-term (12-month duration) PPI use was not associated with changes in serum magnesium levels; however, serum calcium levels declined over time.^[16]^

**Table 6:**
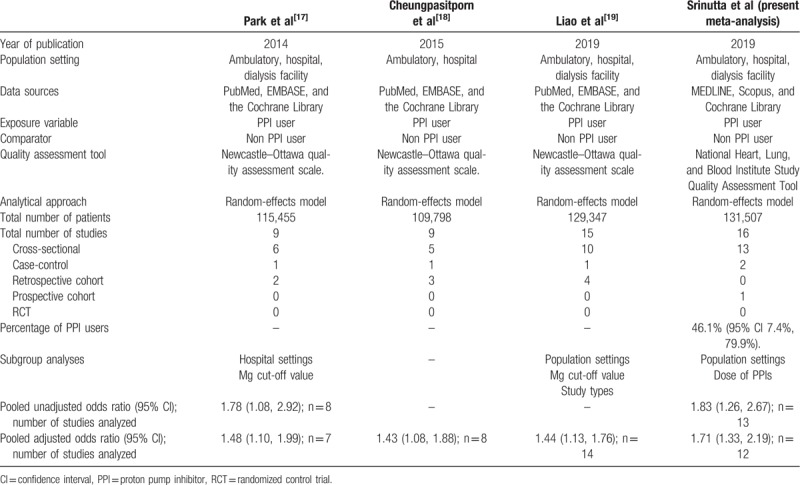
Summary of findings from 4 meta-analyses on the association between use of proton pump inhibitors (PPIs) and hypomagnesemia.

The mechanism of PPI-induced hypomagnesemia is unknown. Current evidence shows that urinary magnesium excretion is not elevated among PPI users, ruling out urinary magnesium losses as a potential mechanism. There is evidence to support intestinal loss or malabsorption of magnesium.^[13,14]^ Furthermore, variant alleles of the *TRPM6*/*TRPM7* genes are associated with subtle intestinal malabsorption and/or persistent urinary losses of magnesium, which might be further aggravated by the use of PPIs in susceptible persons.^[35]^

Our systematic review has several strengths. To the best of our knowledge, this is the first systematic review and meta-analysis of observational studies that explores an association between high-dose PPI (relative to low-dose) and development of hypomagnesemia. We included reports that performed multivariable analyses to account for potential confounders of these associations. However, there are important limitations that should be noted. First, our synthesis of the evidence was limited to observational studies, and in the absence of randomized controlled trials, the cause and effect relation between PPI use and hypomagnesemia remains speculative. Second, there was significant heterogeneity among the individual studies, in terms of clinical settings, study design, indication and dose of PPIs, type of PPIs and duration of use before development of hypomagnesemia. The subgroup analysis linking the PPI dose to hypomagnesemia should be interpreted with caution due to the limited evidence. Furthermore, the definition of hypomagnesemia also varied significantly amongst individual reports. Our analysis is also inconclusive regarding a potential link between the use of PPIs and adverse cardiovascular outcomes, including cardiac arrhythmias mediated by hypomagnesemia.

In conclusion, our systematic review indicates that patients taking PPIs, particularly high-dose PPIs, are at increased risk for developing hypomagnesemia despite significant heterogeneity among individual studies. Hence, we recommend that serum magnesium level be monitor in patients prescribed a PPI long-term, particularly, those prescribed high-dose PPI. Additional post-marketing population-based surveillance studies are needed to further elucidate whether long-term use of PPIs is associated with adverse cardiovascular events, namely hypomagnesemia-induced cardiac arrhythmias.

## Author contributions

**Conceptualization:** Paweena Susantitaphong.

**Data curation:** Thawin Srinutta, Api Chewcharat, Kullaya Takkavatakarn.

**Formal analysis:** Paweena Susantitaphong.

**Methodology:** Paweena Susantitaphong.

**Software:** Paweena Susantitaphong.

**Supervision:** Kearkiat Praditpornsilpa, Somchai Eiam-Ong, Bertrand L. Jaber, Paweena Susantitaphong.

**Validation:** Thawin Srinutta, Api Chewcharat, Kullaya Takkavatakarn, Paweena Susantitaphong.

**Writing – original draft:** Thawin Srinutta, Somchai Eiam-Ong, Bertrand L. Jaber, Paweena Susantitaphong.

**Writing – review and editing:** Thawin Srinutta, Api Chewcharat, Kullaya Takkavatakarn, Kearkiat Praditpornsilpa, Somchai Eiam-Ong, Bertrand L. Jaber, Paweena Susantitaphong.

Paweena Susantitaphong orcid: 0000-0001-9813-9219.

## Supplementary Material

Supplemental Digital Content
